# FOXL2, GATA4, and SMAD3 Co-Operatively Modulate Gene Expression, Cell Viability and Apoptosis in Ovarian Granulosa Cell Tumor Cells

**DOI:** 10.1371/journal.pone.0085545

**Published:** 2014-01-09

**Authors:** Mikko Anttonen, Marjut Pihlajoki, Noora Andersson, Adrien Georges, David L'Hôte, Sanna Vattulainen, Anniina Färkkilä, Leila Unkila-Kallio, Reiner A. Veitia, Markku Heikinheimo

**Affiliations:** 1 Children's Hospital, Pediatric Research Center, University of Helsinki and Helsinki University Central Hospital, Helsinki, Finland; 2 Department of Obstetrics and Gynecology, University of Helsinki and Helsinki University Central Hospital, Helsinki, Finland; 3 Programme de Pathologie Moléculaire et Cellulaire, Institut Jacques Monod, UMR 7592 CNRS-Université Paris Diderot, Paris, France; 4 Department of Pediatrics, Washington University School of Medicine, St Louis, Missouri, United States of America; Baylor College of Medicine, United States of America

## Abstract

Aberrant ovarian granulosa cell proliferation and apoptosis may lead to granulosa cell tumors (GCT), the pathogenesis of which involves transcription factors GATA4, FOXL2, and SMAD3. *FOXL2* gene harbors a point mutation (C134W) in a vast majority of GCTs. GATA4 is abundantly expressed in GCTs and its expression correlates with poor prognosis. The TGF-β mediator SMAD3 promotes GCT cell survival through NF-κB activation, and interacts with FOXL2. Here, we find that the expression patterns of these factors overlap in the normal human ovary and 90 GCTs, and positively correlate with each other and with their mutual target gene *CCND2*, which is a key factor for granulosa cell proliferation. We have explored the molecular interactions of FOXL2, GATA4, and SMAD3 and their roles in the regulation of *CCND2* using co-immunoprecipitation, promoter transactivation, and cell viability assays in human GCT cells. We found that not only SMAD3, but also GATA4 physically interact with both wild type and C134W-mutated FOXL2. GATA4 and SMAD3 synergistically induce a 8-fold increase in *CCND2* promoter transactivation, which is 50% reduced by both FOXL2 types. We confirmed that wild type FOXL2 significantly decreases cell viability. Interestingly, GATA4 and SMAD3 caused a marked reduction of GCT cell apoptosis induced by wild type FOXL2. Thus, the effects of GATA4 and SMAD3 on both cell viability and apoptosis are distinct from those of wild type FOXL2; a perturbation of this balance due to the oncogenic FOXL2 mutation is likely to contribute to GCT pathogenesis.

## Introduction

Granulosa cell tumors (GCTs) are sex cord stromal tumors that represent 3–5% of all ovarian cancers, with two distinct subtypes: the rare juvenile and the more common adult subtype [1]. The overall prognosis is favorable compared to ovarian epithelial cancers, but recurrences occur even decades after primary diagnosis in up to 25–30% of patients, significantly increasing mortality due to the disease [1]–[3]. GCTs are thought to arise from normal granulosa cells of developing follicles and share features of hormonally active granulosa cells [3]. The vast majority of adult type GCTs bear a missense point mutation c.402C>G (codon C134W) in the gene coding for transcription factor *FOXL2* [3]–[5]. The unbiased whole transcriptome analyses on GCT patient samples and cell line suggested that C134W mutated FOXL2 fails to down-regulate genes involved in the control of cell cycle, and to up-regulate (as wild type (wt) FOXL2 does) genes involved in cell death [6], [7]. A mechanistic explanation of the effect of C134W mutation is however still lacking.

FOXL2 is the earliest known marker of ovarian differentiation and a key regulator of ovarian development and function [8], [9]. In *Foxl2^−/−^* mice, the differentiation of granulosa cells is blocked at the primordial follicle stage [9], and a conditional knockout model demonstrated that FOXL2 permanently prevents transdifferentiation of the ovarian granulosa cells into Sertoli-like cells in murine ovaries [8]. In cell models, FOXL2 modulates the expression of key genes in granulosa cell differentiation and steroidogenesis, such as StAR, P450scc, P450CYP17, and aromatase [10], [11]. FOXL2 also regulates cell survival and proliferation in granulosa cell models, inhibiting cell cycle progression [12], and expression of CCND2, a positive regulator of cell cycle progression required for granulosa cell proliferation. Furthermore, wild-type FOXL2 is able to induce apoptosis in granulosa cells, whereas the C134W form is less effective, and may provide malignant granulosa cells with a survival advantage [13], [14].

Several proteins that interact with FOXL2 have been identified, including DEAD box-containing protein DP103 and transcription factors such as the estrogen receptor ERα, the orphan nuclear receptor SF-1 and SMAD3 (reviewed in [10]). Using yeast two-hybrid screening and co-immunoprecipitation we recently identified 10 novel FOXL2 partners of which some are able to repress FOXL2 transcriptional activity irrespective of the promoter [13]. Interestingly, CREM-τ2α, which acted as a repressor on most promoters, increased wt FOXL2 activity on two promoters (PTGS2 and CYP19A1), but was unable to increase the activity of the C134W mutant. The partners that increased the pro-apoptotic capacity of wt FOXL2 failed to increase the pro-apoptotic ability of the C134W mutant FOXL2. Furthermore, the anti-apoptotic partners decreased apoptosis induction by both FOXL2 versions [13].

C134W mutated FOXL2 has been shown to inhibit the activin and GDF-9 induction of anti-proliferative follistatin, which may lead to increased cell proliferation and tumor formation [15]. Activin and TGF-β signaling are mediated by SMAD3, an essential regulator of *CCND2* (cyclin D2) promoter [16]. SMAD3-deficiency in mice causes growth arrest at the preantral follicle stage and decreased granulosa cell proliferation and survival [17]. Deficiency of inhibin-α subunit, or SMAD1/5 in mice leads to the GCT formation; in these murine GCTs SMAD3 is up-regulated and activated (phosphorylated) [18]–[20], suggesting a role for SMAD3 in GCT pathogenesis. Furthermore, genetic removal of SMAD3 expression from inhibin-α deficient mice leads to attenuated granulosa cell tumor progression and down-regulated CCND2 expression [18]. In human GCT cell lines, SMAD3 drives cell viability by activating NF-κB, which in turn up-regulates SMAD3 expression; this positive feedback loop activates the ERK1/2 pathway leading to increased GCT cell survival [21].

SMAD3 interacts with the transcription factor GATA4 in murine cell models [22], suggesting a role of GATA4 in the TGF-β/activin signaling pathway. GATA4 plays a key role in normal granulosa cell function [23], [24] and GCTs [25]–[27]; high GATA4 immunoreactivity denotes a more aggressive human GCT with increased recurrence risk [25]. GATA4 can be presumed to act as an anti-apoptotic factor, by protecting human GCT cells from apoptosis induced by e.g. TRAIL (Tumor Necrosis Factor Apoptosis Inducing Ligand) [27], and by activating an essential apoptosis inhibitor *BCL2* in non-human cell models [26], [28]. Furthermore, GATA4 also activates *CCND2* in different cell models [26], [29], [30].

FOXL2, GATA4, and SMAD3 expression patterns overlap in the fetal and adult ovary [25], [31]–[34], but whether FOXL2 and GATA4 interact physically is unknown. We hypothesized that these factors altogether interact and modulate GCT cell viability through the regulation of CCND2, strongly expressed in GCTs [26]. In current study we show that these factors interact physically and functionally on transactivation of *CCND2* promoter. Wt FOXL2 decreased viable GCT cell number while GATA4, SMAD3, or C134W-mutated FOXL2 did not. Moreover, GATA4 inhibits the wt FOXL2-induced apoptosis, whereas GATA4 has no effect on the reduced capability of C134W-mutated FOXL2 to induce apoptosis. Finally, we demonstrate a positive correlation between the expression levels of FOXL2, GATA4, and SMAD3 in GCTs.

## Materials and Methods

### Human tissue samples and the ethics statement

We utilized the previously characterized GCT tissue microarray of quadruple core samples from 78 primary and 12 recurrent GCTs [25]. The c.402C>G (p.C134W) FOXL2 mutation frequency of 90% in our sample series [5] corresponds to other published GCT series [4]. All histological diagnoses were carefully re-evaluated and patient charts were reviewed to obtain clinical data [2], [25]. For controls, we utilized normal ovarian tissue samples from three pre-menopausal women operated upon for cervical cancer. All the patients were diagnosed at 1971–2003; according to Finnish legislation no written or verbal consent was needed from the patients for using the archive tissue samples coupled with clinical data. This approach was approved by the ethical committee of Helsinki University Central Hospital and the National Supervisory Authority for Welfare and Health in Finland as part of GCT research program.

### Immunohistochemistry

GCT tissue microarray and normal ovarian samples were stained as previously described and presented for GATA4 and CCND2 [25], [26], with following primary antibodies: α-GATA4 1∶400 (sc-1237, Santa Cruz Biotechnology, Santa Cruz, CA, USA), α-FOXL2 1∶400 (IMG-3228, Imgenex, San Diego, CA, USA), α-SMAD3 1∶400 (#51–1500, Invitrogen Corporation, Carlsbad, CA, USA), and α-CCND2 1∶1000 (sc-593; Santa Cruz Biotechnology). The GCT stainings were classified into three groups based on the intensity of staining: high representing intermediary or strong intensity in 70% of the cells, intermediate representing moderate or strong intensity in <70% of the cells, and low representing negative or low intensity even if detected in 100% of the cells; scoring of GATA4 staining as described (34), i.e. high for 80–100% positive nuclei, intermediate for 20–80% positive nuclei, and low for 0–20% positive nuclei. Two researchers (M.A. and N.A. or A.F) independently performed the evaluation and disagreements were resolved by a joint review.

### Cell culture and plasmids

The previously established KGN cells (kindly provided by Dr. T. Yanase, Japan [35]), derived from an adult metastatic GCT, harbor the c.402>G mutation in FOXL2 and were cultured in Dulbecco's modified Eagle's medium (DMEM)/Ham's F-12 medium containing 10% FBS and 1% penicillin/streptomycin [35]. Another GCT derived cell line COV434 [36], that has a wild type FOXL2 genotype [5], and COS-7 cells (ATCC, MD, USA) were grown in DMEM containing 10% FBS and 1% penicillin/streptomycin. pGL3-680 bp *CCND2*-luciferase reporter construct was used for promoter transactivation assay [26]. The over-expression plasmids or the corresponding empty vectors used were; pMT2-GATA4-V5 (V5-tag amino acid sequence GKPIPNPLLGLDST), untagged, V5-tagged, or GFP-tagged pcDNA3.1-FOXL2-WT, pcDNA3.1-FOXL2-C134W, pCDNA3.1-SMAD3 and pCMV-β-Gal.

### Co-immunoprecipitation and Western blotting

COS-7 or COV434 cells were lysed 48 h after transfection in 50 mM Tris, 150 mM NaCl, 1 mM EDTA, 1% Triton X-100 (pH 7.6) supplemented with protease inhibitors PMSF 1 mM and Complete mini EDTA-free cocktail (Roche Diagnostics GmbH, Mannheim, Germany) and phosphatase inhibitors (Sigma Aldrich, St Louis, MO, USA). Clarified lysates were used for Western blotting or immunoprecipitated using Anti-V5 Agarose Affinity Gel (Sigma Aldrich, St Louis, MO, USA) according to instructions. Precipitated proteins were eluted in 2x-SDS sample buffer (100 mM Tris pH 6.8, 4% SDS, 20% glycerol and 0.1% bromophenol blue). 100 mM DTT was further added to the samples. Eluates were separated by SDS-PAGE using NuPage Bis-Tris 4–12% Gels (Invitrogen Corporation) and proteins were electrotransfered onto PVDF membranes (Hybond-P, GE Healthcare, Waukesha, WI, USA). Antibodies used for detection were: α-GATA4 1∶1000 (sc-1237, Santa Cruz Biotechnology,), α-FOXL2 1∶1000 (IMG-3228, Imgenex) and α-SMAD3 1∶1000 (#51–1500, Invitrogen Corporation).

### Promoter transactivation assays

KGN cells were seeded 16 hours prior to transfection at a density of 4×10^4^ cells/cm^2^ and transfected using the calcium-phosphate method (Invitrogen Corporation [37]). The biological activity of luciferase reporter construct was assessed by the Dual-Luciferase® Reporter Assay System (Promega, Madison, WI, USA). Relative luciferase units represent the ratio of firefly luciferase activity over Renilla luciferase activity in the samples. Data is presented as mean of three independent experiments.

### Cell viability and apoptosis assays

For apoptosis/cell viability assay, KGN cells were transfected with the overexpression plasmids by electroporation using a Neon transfection system (Invitrogen Corporation) according to the manual using the following settings: 1400V, 20 ms, 1 pulse. The cell viability was assayed using Cell Proliferation Reagent WST-1 kit (Roche) after 48 h in culture as instructed by manufacturer. Caspase 3/7 activation was measured using Caspase-Glo® 3/7 Assays (Promega) 24 h after transfection following the instructions.

### Data analysis

The immunohistochemical expression data was analyzed with Contingency tabling (2×2) and chi-square or Fisher's exact tests. Cox proportional hazards model was used according to the methodology. The cell culture data was analyzed with oneway ANOVA. The analyses were carried out with JMP® 7.0.1 (SAS Institute Inc., Cary, NC, USA) software; P<0.05 was considered significant. The data represents the means of values obtained from 3 to 6 biologically independent replicates ± SEM.

## Results

### FOXL2, GATA4, and SMAD3 physically interact with each other

**Figure 1:**
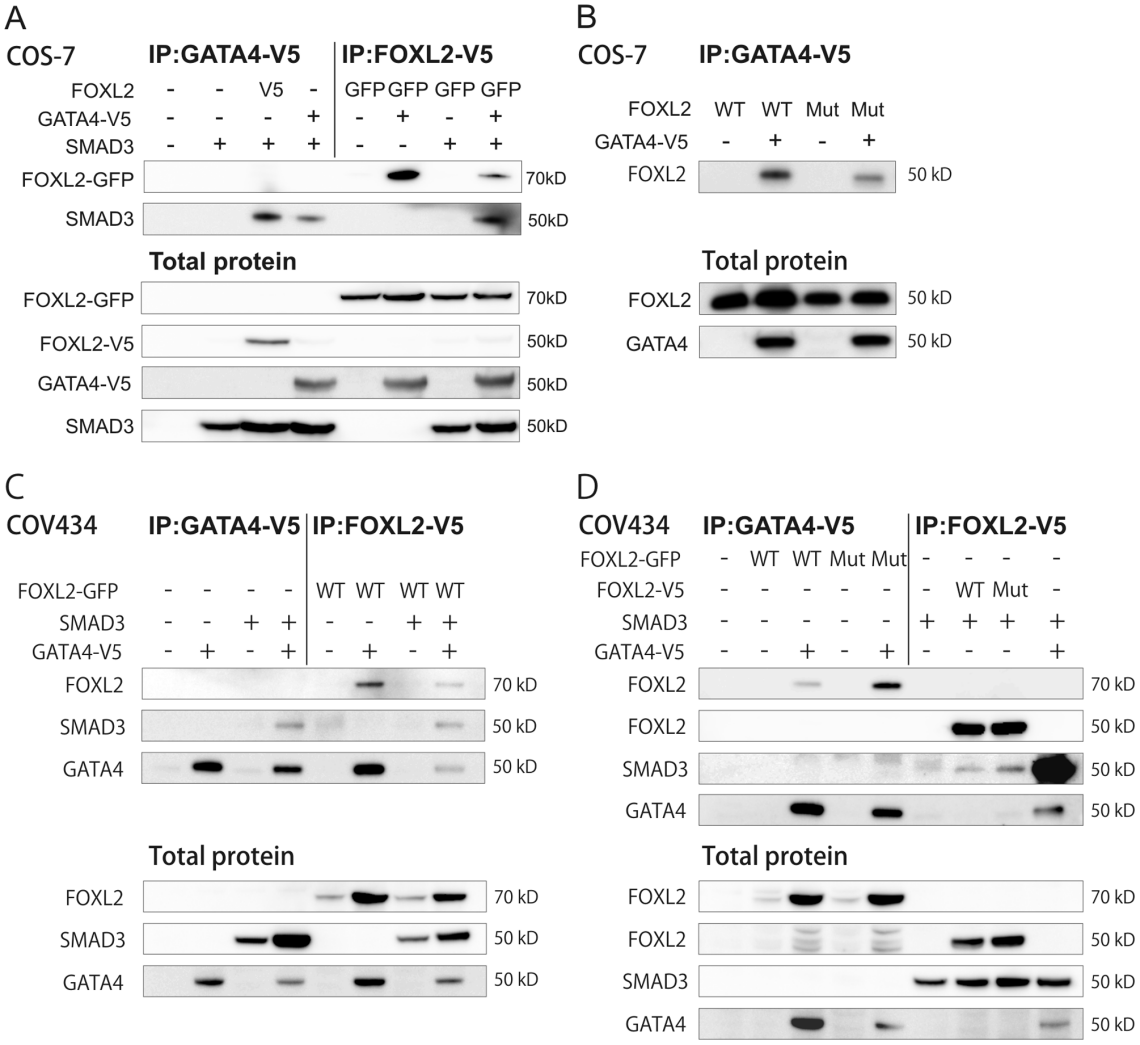
FOXL2, GATA4, and SMAD3 interaction in the transcription complex. Both COS-7 and COV434 cells were transfected for 48 h. All cell lysates were immunoprecipitated using V5 epitope. A) COS-7 cells transfected with V5 tagged GATA4, V5 or GFP tagged wild type (WT) FOXL2 or SMAD3 overexpression vectors as indicated. B) COS-7 cells transfected with V5 tagged GATA4 and untagged wild type (WT) or C134W mutated (MUT) FOXL2 overexpression vectors. C) COV434 cells transfected with V5 tagged GATA4, GFP tagged wild type FOXL2 or SMAD3 overexpression vectors. D) COV434 cells transfected with V5 tagged GATA4, SMAD3 and wild type or C134W mutated FOXL2 overexpression vectors. Immunoprecipitated proteins were detected using antibodies against FOXL2, SMAD3 and GATA4. Total proteins are shown as controls for transfections and were detected using antibodies against FOXL2, GATA4 and SMAD3. Similar results were obtained in at least three independent experiments.

Whereas FOXL2 and GATA4 have previously been shown to co-immunoprecipitate with SMAD3 [15], [22], [38], the interaction of GATA4 and FOXL2 remains unaddressed. To investigate the interactions between FOXL2, GATA4, and SMAD3, we transiently overexpressed V5-tagged GATA4 with SMAD3 and either untagged, V5- or GFP-tagged wt FOXL2 in COS-7 and COV434 cells. Unfortunately, IP experiments were unsuccessful in KGN cells probably due to the low endogenous expression of GATA4, FOXL2 and SMAD3 and poor transfectability. In addition, KGN cells express both wt and mutant FOXL2 protein, which had not allowed testing of interactions of these two variant proteins and other factors. Protein complexes were immunoprecipitated using the V5 epitope. Both wt FOXL2 and SMAD3 co-immunoprecipitated with GATA4 when overexpressed either separately or simultaneously in both cell lines (Figure 1 A and C). To explore the effect of FOXL2 C134W mutation on the interactions between these transcription factors, we next over-expressed V5-tagged GATA4 and SMAD3 with wt and C134W-FOXL2 in COS-7 and COV434 cells. Like wt FOXL2, the C134W-mutated form also co-immunoprecipitated with GATA4 and SMAD3 in both cell lines (Figure 1B and D), showing that a drastic loss of interaction with these factors is not the cause of GCT. Conversely, mutant FOXL2 appears more strongly associated to GATA4 than wt FOXL2 in COV434 cells. Such difference does not appear in COS-7 cells, suggesting that cell type dependent factors may induce a stronger association of C134W FOXL2 to GATA4. Interestingly, the expression of FOXL2 was much stronger in COV434 cells when GATA4 was co-expressed, suggesting that GATA4 may help in stabilizing FOXL2. However, the actual binding affinities cannot be defined based on the immunoprecipitation and western blot analyses.

### GATA4 and SMAD3 synergistically activate CCND2 promoter, and FOXL2 attenuates the CCND2 promoter activation

**Figure 2:**
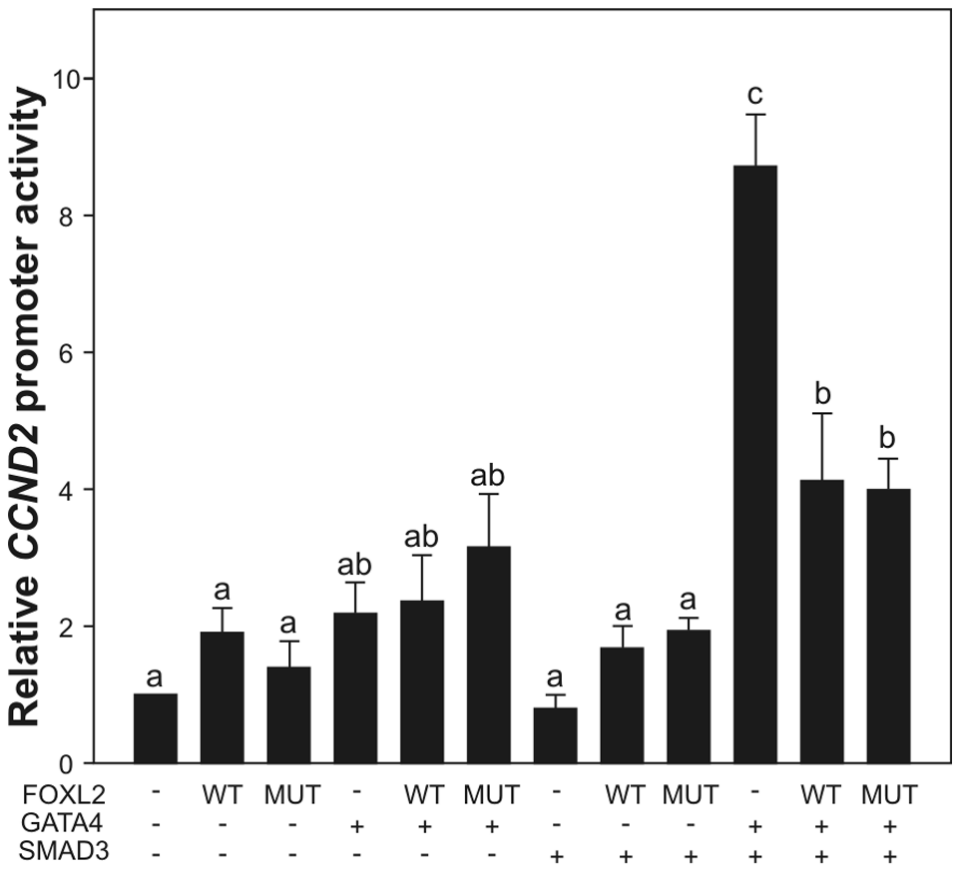
Transactivation of *CCND2* promoter is regulated by FOXL2, GATA4, and SMAD3. Relative luciferase activity was measured in KGN cells after transfection with *CCND2* luciferase promoter construct together with wild type or C134W-mutated FOXL2 in the presence or absence of GATA4 and SMAD3 over-expression. *CCND2* promoter activity was measured 24 h after transfection; at least three independent experiments were performed in triplicate. Bars not connected by the same letter are significantly different. P<0.05.

GATA4 and SMAD3 expression levels have been shown to correlate with that of *CCND2* in GCTs (this study and [26]), and GATA4 and SMAD3 are known to activate *CCND2* promoter [16], [26]. Herein, we overexpressed wt and mutated FOXL2, GATA4, and SMAD3 in KGN cells, and measured *CCND2* promoter activity. The KGN cells express both the C134W-mutated and wt FOXL2 [5], and GATA4 and SMAD3 endogenously. SMAD3 and GATA4 together synergistically increased the *CCND2* promoter activity by 8-fold compared to control (Figure 2). Both FOXL2 forms attenuated the SMAD3/GATA4-induced promoter activity by approximately 50% (Figure 2). In COS-7 cells, both FOXL2 forms also significantly reduced GATA4-induced *CCND2* promoter activity (data not shown).

### Effects of FOXL2, GATA4, and SMAD3 on cell viability and apoptosis in GCT cells

**Figure 3:**
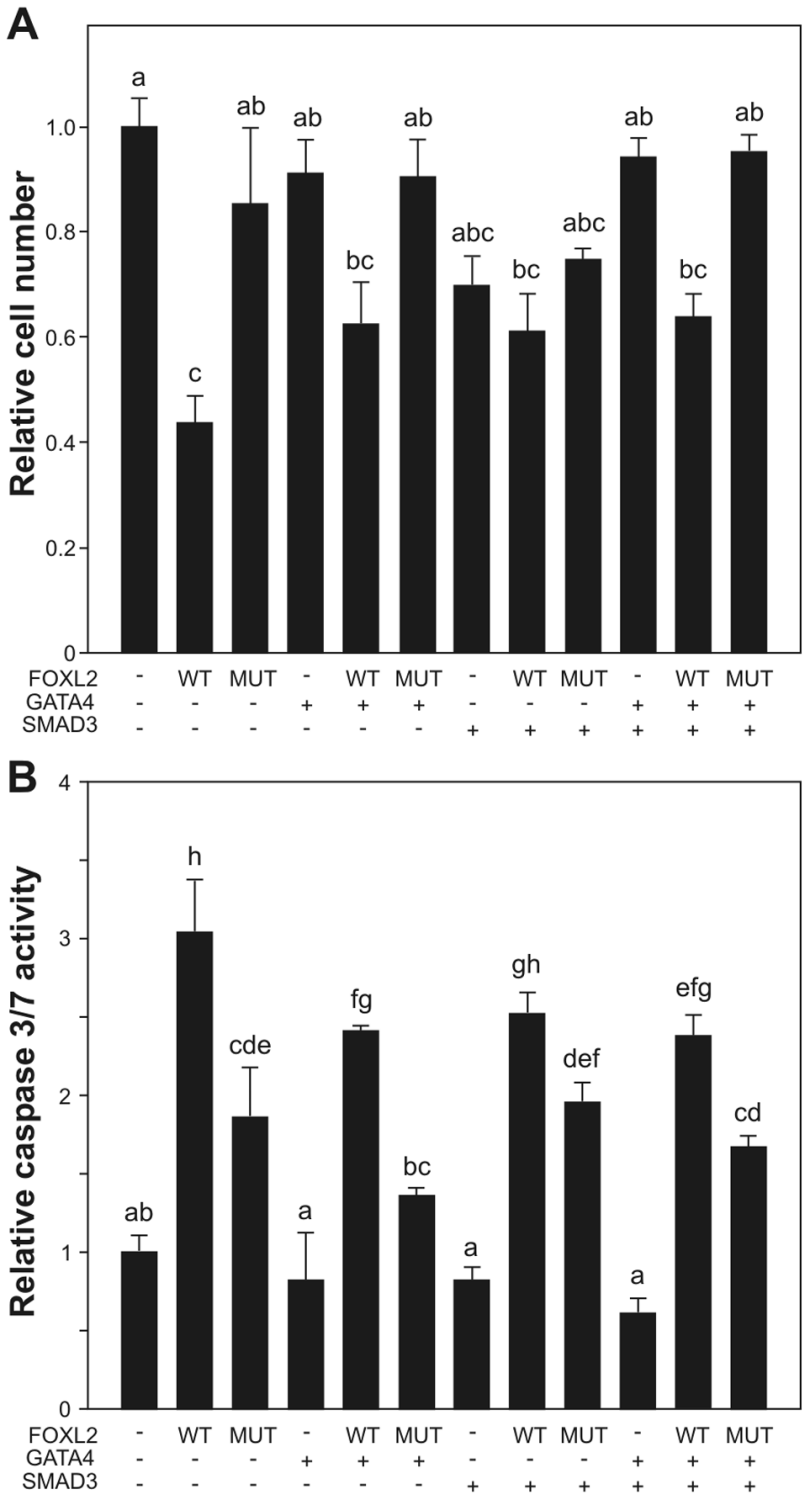
Wt FOXL2 reduces viable cell number, and GATA4 protects GCT cells from wt FOXL2 induced apoptosis. KGN cells were transfected with wild type FOXL2, C134W mutated FOXL2, GATA4, and SMAD3 expression plasmids. A) The cell viability was measured 48 h after transfection. B) The activated caspase 3/7 was measured 24 h after transfection. Viable cell number and Caspase 3/7 activity are presented relative to control transfection as the mean ± SEM of at least three independent experiments performed in triplicate. Bars not connected by the same letter are significantly different. P<0.05.

Given the effects of GATA4, SMAD3, and both FOXL2 types on the *CCND2* promoter, we next analyzed the effect of these factors on cell viability by overexpressing them in KGN cells using WST-1 cell viability assay 48 h after transfection. Wt FOXL2 overexpression alone or together with GATA4 and/or SMAD3 significantly reduced the cell viability compared to control, while overexpression of the mutated FOXL2 alone or together with GATA4 and/or SMAD3 had no significant effect on the relative cell number (Figure 3A). Mutated FOXL2 had distinct effects on cell viability compared to wt FOXL2 when overexpressed with GATA4 and SMAD3.

GATA4 has been shown to inhibit apoptosis [27], whereas FOXL2 has been shown to induce apoptosis [13], [14]. To study whether GATA4 and/or SMAD3 modulate the FOXL2 induced apoptosis, KGN cells were transfected with wt and C134W versions of FOXL2, GATA4, and SMAD3 overexpression vectors, and activation of caspase3/7 was measured as an indicator of apoptotic activity 24 h after transfection (Figure 3B). Similarly to previous findings [13], [14], wt FOXL2 induced a 3-fold increase in caspase 3/7 activity (p = <0.0001), whereas C134W mutated FOXL2 showed significantly weaker effect (p = <0.0001). Overexpression of GATA4 or SMAD3 alone or together had no effect on caspase3/7 activity. GATA4, but not SMAD3, significantly reduced wild type FOXL2-induced apoptosis (p = 0.0194), but had no significant effect on C134W FOXL2-induced caspase 3/7 activation (Figure 3B).

### FOXL2, GATA4, and SMAD3 have overlapping expression patterns with each other and with CCND2 in GCTs

**Figure 4:**
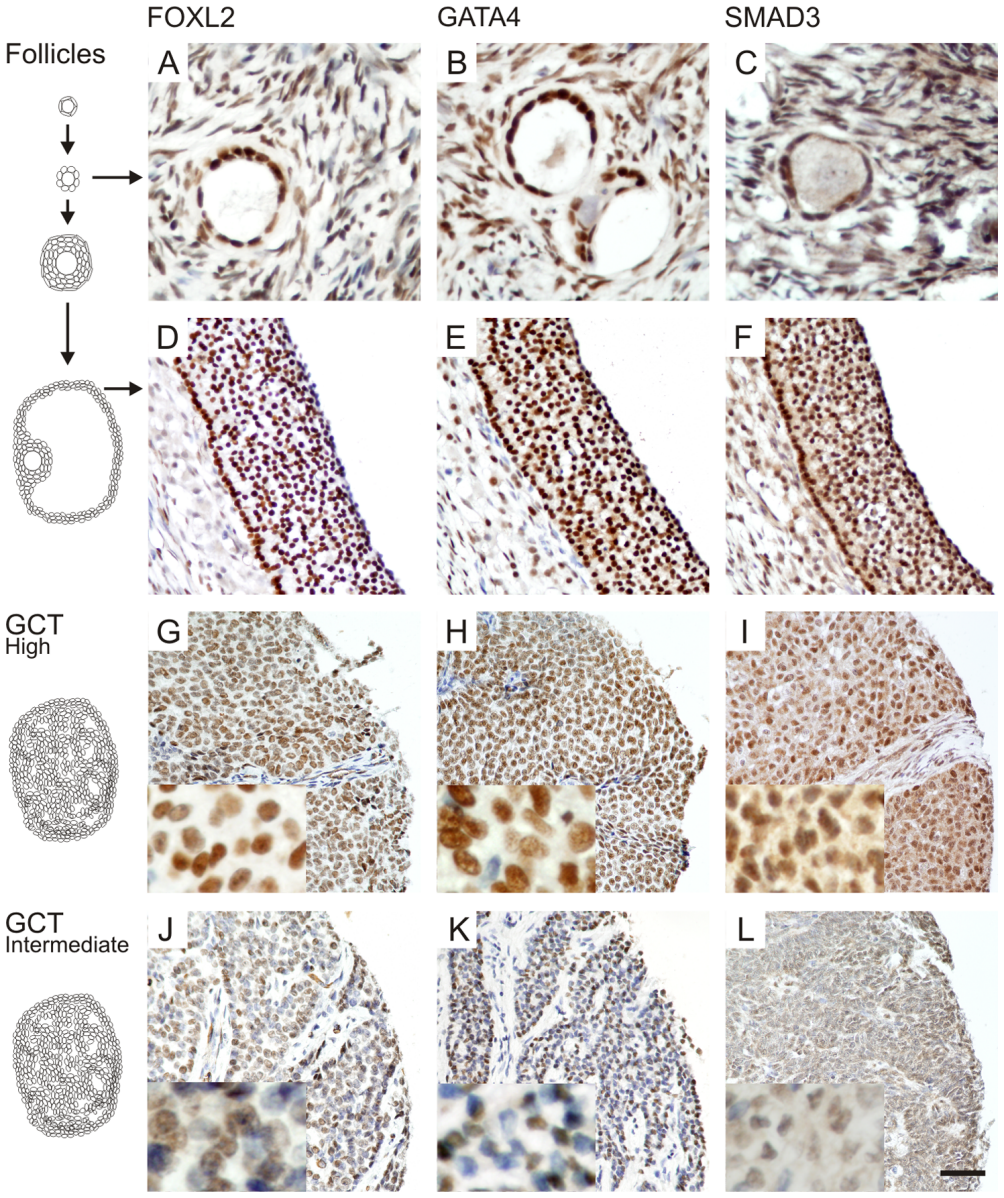
FOXL2, GATA4, and SMAD3 proteins are expressed in normal follicles and GCTs. Proliferating granulosa cells of primary (A–C) and antral (D–F) follicles express high levels of the transcription factors FOXL2, GATA4 and SMAD3. For GCTs, High- (G–I) and intermediate-level (J–L) expressing examples are shown. FOXL2 and GATA4 expression localized into the nucleus, while low levels of SMAD3 expression was also detected in the cytoplasm. Higher magnifications are shown in insets. Scale bar  = 100 µm.

Finally, we analyzed the spatiotemporal protein expression patterns of FOXL2, GATA4, and SMAD3 in normal ovaries and in a tissue microarray containing 90 GCT samples. Granulosa cells of proliferating follicles from primary stage to large antral ovulatory follicles demonstrated nuclear expression of transcription factors FOXL2, GATA4, and SMAD3 (Figure 4A–F); SMAD3 protein was also present in the cytoplasm. Previously, we have shown that CCND2 is similarly expressed in normal granulosa cells of primary and antral follicles as well as in GCTs [26]; expression patterns, representative images, and scoring results for GATA4 and CCND2 have been previously published [25], [26]. For all these four factors the staining intensities of GCTs were classified into low-, intermediate- and high-level groups. The majority of GCTs expressed high or intermediate levels of FOXL2, GATA4, SMAD3 (Figure 4G–L, Table 1), and CCND2 (Table 1). The high expression patterns between the four factors overlapped, which lends credence to their physical interactions demonstrated above (Table 2); in short, FOXL2 and GATA4 protein expression patterns positively correlated with each other and with that of SMAD3. GATA4 and SMAD3, but not FOXL2 correlated with CCND2.

**Table 1:** Protein expression patterns in 78 primary and 12 recurrent granulosa cell tumors.

	Staining intensities n (%)				
	GATA4^1^	FOXL2	SMAD3	CCND2^1^	
High	39 (43%)	33 (37%)	36 (41%)	36 (40%)	
Intermediate	42 (47%)	52 (58%)	43 (50%)	39 (43%)	
Low	9 (10%)	5 (5%)	8 (9%)	15 (17%)	
**Total**	90	90	87	90	

^1^ Expression data for the primary GCTs has been previously reported for GATA4 [25], and for CCND2 [26].

**Table 2:** Correlation of protein expression patterns in 78 primary and 12 recurrent granulosa cell tumors.

	Staining correlations^1^				
	GATA4	FOXL2	SMAD3	CCND2	
GATA4		**+** (0.0031)	**+** (0.0003)	**+** (<0.0001)	
FOXL2	**+** (0.0031)		**+** (0.0119)	ns	
SMAD3	**+** (0.0003)	**+** (0.0119)		**+** (0.0241)	
CCND2	**+** (<0.0001)	ns	**+** (0.0241)		

^1^ Intermediate and low staining were grouped together in the analysis; + indicates positive correlation with p<0.05 (p values presented), ns =  not significant, blank =  same antigen.

The protein expression patterns of these four factors did not correlate with any analyzed clinicopathological parameters of the primary tumors, i.e., age and menopause status of the patient at diagnosis, clinical stage, size of the tumor, tumor subtype, nuclear atypia, and mitotic index [2], [25]. In subgroup analyses of larger and more aggressive GCTs, however, the interrelations of FOXL2/GATA4/SMAD3 expressions were altered. The positive correlations of FOXL2 and GATA4 with each other and with SMAD3 were lost in tumors over 10 cm in diameter (n = 35) (p>0.05), and the positive correlation between FOXL2 and GATA4 was absent in the primary GCTs that had recurred (n = 19) (p>0.05). Nevertheless, in Cox proportional hazards model, high FOXL2 associated with an increased 5 years risk of recurrence (RR 8.7, 95%CI 1.4–167.0, p = 0.018) and low FOXL2 expression with low risk for tumor recurrence in 5 years (RR 0.12, 95%CI 0.006–0.71, p = 0.018).

## Discussion

The ability of tumor cells to evade apoptosis and to proliferate excessively is a general property of cancer. FOXL2 is a potential activator of granulosa cell apoptosis [14], as well as an inhibitor of the cell cycle [12]. The balanced regulation of granulosa cell proliferation and apoptosis is perturbed by the FOXL2 C134W mutation [3], that fails to down-regulate genes involved in the control of cell cycle and to up-regulate genes involved in apoptosis [6], [7]. In contrast, GATA4 functions as an antiapoptotic factor in GCT cell models and high GATA4 expression in GCTs is associated with aggressive disease [25]–[27]. Moreover, SMAD3 positively regulate the GCT cell proliferation by e.g. activating the ERK1/2 signaling pathway [17], [21]. The spatiotemporal expression patterns of FOXL2, GATA4, and SMAD3 coincide within the somatic cells of the fetal and adult ovary and we now find that human GCTs typically co-express FOXL2, GATA4, and SMAD3.

Based on previous and present data, we hypothesized that FOXL2, GATA4, and SMAD3 physically interact. Coherently, co-immunoprecipitation data show the previously unknown binding of GATA4 to FOXL2, and that SMAD3 interacts with both GATA4 and FOXL2. The immunoprecipitation data do not provide direct evidence that the three transcription factors are part of the same macromolecular transcription complex but rather proves that they have binary physical interactions with each other, congruent with their co-expression in both normal granulosa cells and GCTs. It is conceivable that the presence of FOXL2 disrupts the interaction between GATA4 and SMAD3 and therefore their synergistic regulation of key target promoters. Altogether, the lack of positive expression correlations of FOXL2 and GATA4 in the more aggressive GCTs suggests that imbalances in FOXL2-GATA4-SMAD3 expression might increase the growth potential of GCTs, leading to a more aggressive tumor behavior. The results are also coherent with previous findings on high GATA4 or FOXL2 expression in the primary tumor associated with risk of recurrence [25], [39].

The mouse *Ccnd2* promoter harbors GATA4 binding sites, and GATA4 binding to the *Ccnd2* promoter is required for murine cardiomyocyte proliferation [30]. GATA4 activates *Ccnd2* in murine GCT cells in transactivation assays [26], whereas FOXL2 has been shown to repress *CCND2* promoter, which contains several consensus binding sites for the family of forkhead transcription factors 5′-[(G/A)(T/C)(C/A)AA(C/T)A]-3′. In KGN cells, neither FOXL2, GATA4, nor SMAD3 over-expression alone could significantly enhance *CCND2* promoter activity, whereas simultaneous over-expression of GATA4 and SMAD3 induced a synergistic activation of the promoter. Attenuation of the promoter activation by both wt and C134W-mutated FOXL2 suggests, along with the immunoprecipitation data, that failure in the FOXL2-GATA4-SMAD3 physical and/or functional interactions with *CCND2* promoter cannot fully explain how the FOXL2-C134W mutation contributes to GCT pathogenesis. In rat granulosa cells, activation of *Ccnd2* promoter requires FSH-induced release of FOXO1 from the promoter and positive signals from activin-SMAD2/3 pathway; consensus SMAD3 response elements 5′-[CAGACA]-3′ have been identified on the rat *Ccnd2* promoter. Our findings suggest that these signals involve GATA4/SMAD3 synergy, supporting the role of GATA4 in the proliferative TGF-β/activin signaling in granulosa cells [22]. Given the positively correlated protein expression patterns of GATA4-SMAD3-CCND2 in GCTs, the data suggest that GATA4-SMAD3 co-operation is essential for CCND2 expression and sustained proliferation of malignant granulosa cells.

Previously, endogenous GATA4 has been shown to inhibit apoptosis in GCT cells, while the dominant negative form of GATA4 induced basal apoptosis [27]. Wild type and the C134W-mutated FOXL2 have differential abilities to induce apoptosis [13], [14], and we now demonstrate that GATA4 is able to protect KGN cells from wt FOXL2-induced apoptosis in overexpression conditions, further supporting the anti-apoptotic role of GATA4. Our cell viability assay showed that wild type FOXL2 significantly attenuated the GCT cell viability, while mutated FOXL2 lacked this ability. This is in line with our previous data demonstrating that mutated FOXL2 fails to interact with some of its pro-apoptotic partner which promotes cell viability [13], altogether indicating that mutation in *FOXL2* gene gives the GCT cells a growth advantage, probably not directly through CCND2 regulation but by decreasing sensitivity to apoptosis.

In conclusion, we demonstrate a previously unknown interaction between FOXL2 and GATA4, together with SMAD3, and these transcription factors can modulate the promoter activity of the key target genes involved in GCT cell proliferation and survival, such as *CCND2*. Moreover, the data further strengthens the role of SMAD3 in human GCT pathogenesis, as previously suggested by studies in GCT mouse models and human GCT cell line. GATA4 and SMAD3 exhibit distinct effects compared to wild type FOXL2 in the regulation of cell survival, whereas these factors do not modulate decreased ability of C134W-FOXL2 to induce apoptosis. This links GATA4 and SMAD3 into the balanced regulation of granulosa cell viability and apoptosis, which is abrogated by C134W-mutated FOXL2 in the GCTs.
